# Factors Influencing Depression and Mental Distress Related to COVID-19 Among University Students in China: Online Cross-sectional Mediation Study

**DOI:** 10.2196/22705

**Published:** 2021-02-22

**Authors:** Yanqiu Yu, Rui She, Sitong Luo, Meiqi Xin, Lijuan Li, Suhua Wang, Le Ma, Fangbiao Tao, Jianxin Zhang, Junfeng Zhao, Liping Li, Dongsheng Hu, Guohua Zhang, Jing Gu, Danhua Lin, Hongmei Wang, Yong Cai, Zhaofen Wang, Hua You, Guoqing Hu, Joseph Tak-Fai Lau

**Affiliations:** 1 Centre for Health Behaviours Research Jockey Club School of Public Health and Primary Care The Chinese University of Hong Kong Hong Kong Hong Kong; 2 School of Public Health Dali University Kunming China; 3 Graduate School of Baotou Medical College Baotou Medical College Baotou China; 4 Health Science Center School of Public Health Xi’an Jiaotong University Xi’an China; 5 Department of Maternal, Child and Adolescent Health School of Public Health Hefei China; 6 School of Public Health Sichuan University Chengdu China; 7 Department of Psychology School of Education Henan University Kaifeng China; 8 Shantou Medical College Shantou China; 9 Department of Epidemiology and Health Statistics College of Public Health Zhengzhou University Zhengzhou China; 10 Department of Biostatistics and Epidemiology School of Public Health Shenzhen University Health Science Center Shenzhen China; 11 Department of Psychology School of Psychiatry Wenzhou Medical University Wenzhou China; 12 Department of Medical Statistics School of Public Health Sun Yat-Sen University Guangzhou China; 13 Faculty of Psychology Beijing Normal University Beijing China; 14 School of Public Health Zhejiang University School of Medicine Hangzhou China; 15 School of Public Health Shanghai Jiao Tong University School of Medicine Shanghai China; 16 Public Health Department Qinghai University Medical College Xining China; 17 Department of Social Medicine and Health Education School of Public Health Nanjing Medical University Nanjing China; 18 Department of Epidemiology of Health Statistics Xiangya School of Public Health Central South University Changsha China

**Keywords:** COVID-19, depression, mental distress, psychological responses, mediation, China, online survey

## Abstract

**Background:**

The COVID-19 epidemic may elevate mental distress and depressive symptoms in various populations in China.

**Objective:**

This study investigates the levels of depression and mental distress due to COVID-19, and the associations between cognitive, behavioral, and psychosocial factors, and depression and mental distress due to COVID-19 among university students in China.

**Methods:**

A large-scale online cross-sectional study (16 cities in 13 provinces) was conducted among university students from February 1 to 10, 2020, in China; 23,863 valid questionnaires were returned. The Patient Health Questionnaire-9 was used to assess depression. Structural equation modeling was performed to test mediation and suppression effects.

**Results:**

Of the 23,863 participants, 47.1% (n=11,235) reported high or very high levels of one or more types of mental distress due to COVID-19; 39.1% (n=9326) showed mild to severe depression. Mental distress due to COVID-19 was positively associated with depression. All but one factor (perceived infection risks, perceived chance of controlling the epidemic, staying at home, contacted people from Wuhan, and perceived discrimination) were significantly associated with mental distress due to COVID-19 and depression. Mental distress due to COVID-19 partially mediated and suppressed the associations between some of the studied factors and depression (effect size of 6.0%-79.5%).

**Conclusions:**

Both mental distress due to COVID-19 and depression were prevalent among university students in China; the former may have increased the prevalence of the latter. The studied cognitive, behavioral, and psychosocial factors related to COVID-19 may directly or indirectly (via mental distress due to COVID-19) affect depression. Interventions to modify such factors may reduce mental distress and depressive symptoms during the COVID-19 epidemic.

## Introduction

COVID-19 started in Wuhan, China in December 2019 [1] and was declared a pandemic on March 11, 2020 [2]. As of January 10, 2021, there were 87,364 deaths and over 89.4 million confirmed cases in China and overseas [3]. In China, the initial phase of the COVID-19 outbreak induced numerous stressors, as it impacted almost every aspect of daily life, from work and entertainment to service use and social interactions [4]. For instance, entry to and exit from Wuhan and many cities and regions have been prohibited since the Chinese Lunar New Year (CNY). Other personal and public control measures include closure of offices and public areas (eg, shopping areas, offices, and restaurants), massive quarantines, staying at home for a long period of time, suspension of school classes, and cancellation of events [4]. It is important to study the negative psychological responses potentially caused by the COVID-19 epidemic, as previous studies have reported high prevalence of depression and other mental health problems in various populations during the 2003 severe acute respiratory syndrome (SARS) epidemic, the 2009 novel influenza A (H1N1) pandemic, and the 2014-2016 Ebola outbreak [5-8]. Mental health problems have significant public health implications, as they affect the public’s use of measures for prevention [9]. The study of negative psychological responses and associated factors related to the COVID-19 outbreak in China allows global public health and mental health workers to assess related service demands and design effective interventions.

Although a number of studies have looked at the factors of depression during the COVID-19 period, fewer studies were conducted in the initial phase of the outbreak, which started after the Chinese government announced the disease’s person-to-person transmission property on January 20, 2020, and started the first controversial Wuhan lockdown 3 days after [10]. To our knowledge, no similar studies were conducted outside China around that period of time, as COVID-19 had not become a pandemic until March and gained less attention outside China in early February 2020. Psychological responses are context specific. The initial phase of the COVID-19 outbreak (in terms of the first few weeks) in the world meant uncertainties and a lack of information. It is imperative to document the community responses at the initial stage of new emerging infectious diseases (especially those that eventually become a pandemic) to inform preparations for future outbreaks.

Furthermore, the first COVID-19 outbreak occurred in China during the CNY, which involved high mobility, as billions of people were returning to their hometowns and, hence, created a high risk of spreading the virus to the entire country. Mobile populations have special relevance in this specific time and country context, one of which was university students, as the majority of students were studying in cities away from their hometowns. This study investigates psychological responses of depression and associated factors among university students in China from 10 to 20 days since COVID-19 was believed to involve person-to-person transmission. Our literature search found only four similar university student studies conducted during the same time period [11-14]. Three of them covered only one or two cities and provinces, and the fourth one claimed to involve 29 provinces but had a sample size of only 2216, while this study covers 16 cities in 13 provinces (n=23,863). Moreover, this study is population-based, while no sampling frame was mentioned in the four other studies.

Although factors of depression during the COVID-19 period have been widely reported. There are still substantial research gaps that are being filled by this study besides the aforementioned fact that such studies were scarce during the initial weeks. Cognitive, behavioral, and psychosocial factors related to COVID-19 were all found to be potential determinants of depression [15-17]. Although a lot of studies have investigated associations between COVID-19–related cognitive factors and preventive behaviors [18,19], only a few studies have looked at COVID-19–related cognitive factors of depression. For instance, the four Chinese university student studies conducted during the initial outbreak mainly mentioned lifestyle factors such as sleep and psychological attributes such as resilience but not COVID-19 cognitive factors [11-14]. Several studies of the population in China and overseas looked at cognitive factors such as perceived susceptibility and severity, which were positively associated with depression [15,20-23].

According to the fear appeal theory, perceived threat comprises perceived susceptibility and perceived severity [24]. The theory provides a framework to include cognitive factors in this study. Perceived chances for oneself or one’s significant others in contracting a disease (perceived susceptibility) [9] was significantly associated with psychological problems such as depression during the SARS, H1N1, and Ebola outbreaks [5,7,8]. In general, perceived severity of a disease was significantly associated with negative psychological responses [7,8]. The public’s belief that H1N1 could cause severe irreversible bodily damage was associated with mental distress [7]. Anticipation about the likelihood and scale of a potential outbreak reflects perceived severity of the epidemic at the community level. For instance, perceived chances of having large H1N1 and Ebola outbreaks were associated with mental health problems [5,7]. We contended that perceived chance of controlling the COVID-19 epidemic would be positively associated with depression, as such a perception may reduce perceived severity due to potential negative impacts (eg, finance, work, and social relationship).

Misconceptions that H1N1 could be transmitted via some unconfirmed modes of transmission such as waterborne transmissions increased perceived susceptibility, which was associated with mental distress [8]. The association between misconceived mode of COVID-19 transmission and depression was less clear. One study conducted in the general population during the initial phase of COVID-19 in China found that the perceived mode of transmission via droplets increased risk of depression, while perceived transmission via contaminated objects or airborne transmission was nonsignificant. As people were forming their perceptions based on the risk, severity, and mode of transmission during the initial outbreak phase amid uncertainties and a lack of knowledge, their associations with depression might be different from those obtained from subsequent studies conducted during the later phases of the pandemic.

COVID-19–related behavioral factors of depression are also important, as behavioral responses would occur during initial outbreaks of new emerging infectious diseases. An important and unprecedented response was staying at home during the CNY in China, keeping in mind that there were then no clear strict social distancing policies in China, and there was no penalty for going out in most Chinese cities. It is important to understand the level of staying at home during the critical initial outbreak phase, as it had contributed to the control of COVID-19 in China by reducing social contacts. Research has found that quarantine was positively associated with depression [25], but staying at home during the holidays is different from quarantine and working from home. Other studies found that social distancing, or more precisely compliance to social distancing policies, was positively associated with depression [26,27], while nonsignificant results have also been reported (eg, [28]). No study has looked at the association between voluntarily staying at home and depression during the initial COVID-19 period in China. Another important unique behavioral factor was close contacts with people from Wuhan, which was the first and most important epicenter of COVID-19. At the time of the survey, a majority of the COVID-19 cases in China were detected from Wuhan, while many cases detected outside Wuhan were related to visitors of Wuhan [10]. Close contacts with such people was a unique stressor that was investigated only in this study. Choosing to stay at home as a prevention strategy may reduce perceived susceptibility, as it lowers the likelihood of contracting COVID-19. Having closely contacted high-risk people such as those who had travelled to Wuhan may increase perceived susceptibility. Thus, it is essential to look at whether they have associations with depression after adjusting for perceived severity and susceptibility.

Potential psychosocial factors related to COVID-19 may be associated with mental distress at the community level. For instance, perceived discrimination is a risk factor of depression [29] and has been associated with mental distress in SARS research [30]. Health care workers and patients who recovered from SARS were discriminated against by the public [31]. As of January 10, 2021, there were 50,340 confirmed COVID-19 cases in Wuhan and 17,809 in the rest of the Hubei Province (where Wuhan is located) [32]. Five million people had traveled out of Wuhan during the CNY [33]. Those who had visited Wuhan or Hubei might have encountered discrimination. In addition, people at risk of contracting the virus were prone to encountering discrimination (eg, hospital workers and their close contacts, and family members, coworkers, and neighbors of infected cases). No study has looked at COVID-19–related discrimination and its potential effect on depression during the COVID-19 outbreak in China.

Mental distress due to COVID-19 (eg, panic, anxiety, and emotional agitation) is potentially associated with depression. It is understood that prevalence of depression among university students prior to the COVID-19 outbreak was not low; not all depressive symptoms were caused by COVID-19 although the pandemic could have inflated the risk of depression. This study thus has the novelty of measuring both general depressive symptoms and the level of self-reported mental distress directly attributed to COVID-19, based on a scale used in understanding mental distress due to SARS and H1N1 [7,34,35]. It is imperative to understand the associations between cognitive, behavioral, and psychosocial factors, and both mental distress due to COVID-19 and depressive symptoms, which were tested in this study.

Another research gap is that few studies have looked at the mechanisms between COVID-19–related factors and depression during the pandemic period. According to the common sense model of illness representation, cognitive perceptions, how a person feels about a disease (ie, emotional representation), and their coping responses such as behavioral responses would determine health outcomes, including mental health status [36,37]. Thus, it is contended that cognitive, behavioral, and psychosocial factors, and mental distress due to COVID-19 would be positively associated with depression. Furthermore, this study tested the mediation hypothesis that emotional responses (ie, mental distress due to COVID-19) mediated between the studied cognitive factors (eg, perceived bodily damages and perceived infection risk of COVID-19), behavioral factors (eg, staying at home and having close contacts with people who visited Wuhan), and psychosocial factors (eg, perceived discrimination related to COVID-19), and depressive symptoms. No study has looked at such mediations, and thus, this study contributes to the literature of mental distress during the initial COVID-19 period.

This study investigates the level of depression among 23,863 university students of 26 universities located at 16 cities in 13 provinces of China 10 days after the official recognition of person-to-person transmission by the Chinese government and during the 8th to 17th days of the CNY in China, which was the initial rising phase of COVID-19. Based on the literature search, besides background and contextual factors, this study investigates the associations between the following factors and both mental distress due to COVID-19 and depression: *cognitive factors* (ie, misconceptions about modes of transmission; perceived risks of contracting COVID-19 for self, family members, and classmates; perceived permanent bodily damages of COVID-19; and perceived chance in controlling the epidemic in China in the coming 6 months), *behavioral factors* (ie, staying at home behavior during the CNY and close contacts with people who had visited Wuhan before the CNY), and *psychosocial factors* (ie, perceived discrimination) related to COVID-19. We further tested the hypothesis that mental distress due to COVID-19 would mediate between the aforementioned cognitive, behavioral, and psychosocial factors, and depression. The literature has not reported similar studies.

## Methods

### Participants and Procedure

This cross-sectional study was conducted during the 8th to 17th days of the CNY (February 1-10, 2020). Data were collected from 26 universities of 16 cities in 13 out of the 32 provinces, municipalities, and autonomous regions in the country. A total of 681 classes were sampled by convenience within a number of faculties (arts, sciences, social sciences, engineering, medicine and public health, and others). The median number of students selected per university was 1165 (IQR 2271). All students of the selected classes were sent a QR (Quick Response) code through Wechat to access an anonymous online questionnaire that took about 10-15 minutes to complete. They were informed about the study’s background, anonymity, restriction to academic use, and that return of the completed questionnaire implied informed consent. A lucky draw gave out five prizes of ¥50-¥200 (about US $7-$28) per city, while half of the students randomly received a symbolic CNY *lucky money* (red pocket) of ¥1 (about US $ 0.15).

A total of 36,560 invitations were sent out; 25,647 completed questionnaires were returned, 1197 (4.7%) of which did not pass the consistency checks and were excluded from data analysis together with 47 (0.2%) others who were diagnosed COVID-19 positive, 515 (2.0%) who were quarantined, and 25 (0.1%) who were outside mainland China. The effective sample size was 23,863 (93.0%). The response rate was 70.2% (25,647/36,560).

The study was approved by the Survey and Behavioral Research Ethics Committee of the Chinese University of Hong Kong (No. SBRE-19-400).

### Measures

#### Personal Background

Personal background information included sociodemographic data (ie, sex), school-related information (ie, grades and faculty), and self-reported physical health status.

#### Contextual Factors

Contextual factors included living arrangement during the CNY (ie, whether staying in their university’s city), whether staying with their families at the time of the survey, whether their localities of stay were *shut down* by the local government during the CNY, and the number of confirmed COVID-19 cases detected in the provinces that the participants’ localities belonged to.

#### Depression

Depression was assessed by using the Patient Health Questionnaire-9 (PHQ-9). It has been validated in Chinese populations and has shown good psychometric properties [38]. The items asked about the frequency that some symptoms occurred during the past 2 weeks; sample items involved “little interest or pleasure in doing things” and “feeling down, depressed, or hopeless.” Each item was rated with a four-point Likert scale (from 0, not at all, to 3, nearly every day). Summative scores of 5, 10, 15, and 20 represent cutoff points for defining mild, moderate, moderately severe, and severe depression, respectively. The Cronbach alpha of the PHQ-9 was .92 in this study.

#### Mental Distress Due to COVID-19

Three items were used to assess the levels of mental distress due to COVID-19 (ie, panic, anxiety, and emotional agitation). The items were rated with a four-point Likert scale (from 1, very low, to 4, very high); higher levels of the summative score indicate higher levels of mental distress due to COVID-19. The summative scale has been used in a number of H1N1 studies [7,8]. The Cronbach alpha was .93 in this study.

#### Cognitive Factors Related to COVID-19

First, the item on *airborne transmission* stated “COVID-19 can be transmitted long distance through air” (responses: 1, yes; 2, no; or 3, uncertain); the responses were recoded into two groups (1, yes, or 0, no or uncertain). Second, three items were used to assess the levels of *perceived infection risk* of COVID-19 in the coming year for oneself, family members, and classmates (responses: from 1, very low, to 4, very high, and 5, do not know or not applicable); the responses were recoded into two groups (1, very high or high, and 0, less than high). The Perceived Infection Risk Indicator was then formed by counting the number of ones (range of 0-3). Third, the item on *perceived permanent bodily damage* stated “COVID-19 will easily cause severe permanent bodily damage” (response: 1, agree; 2, disagree; or 3, do not know); the responses were recoded into two groups (1, agree, and 0, disagree or do not know). Fourth, the item *perceived chance of controlling the COVID-19 epidemic in China* stated “What is the chance that the COVID-19 epidemic will be controlled in China in the coming six months” (responses: from 1, definitely yes, to 6, definitely no, and 7, uncertain); the responses were recoded into two groups (1, definitely yes or very high, and 0, less than very high).

#### Behavioral Factors Related to COVID-19

First, the *staying at home* item assessed the total number of hours spent out during the 7-day CNY (0 hours, 1 hour to 4 hours, 5-10 hours, 11-14 hours, or ≥15 hours). Second, the item *close contacts with people who had visited Wuhan* asked “Have you closely contacted people who had visited Wuhan within the two weeks before CNY?” The response was recoded into two groups (1, yes, or 0, no or do not know).

#### Perceived Discrimination

One item assessed the level of perceived discrimination encountered due to COVID-19 (from 1, very low, to 4, very high).

### Statistical Analysis

The summative score of the PHQ-9 was used as the continuous dependent variable. The associations between the background personal variables, contextual variables, and depression were analyzed by simple regression models; Spearman correlation coefficients were derived to assess the correlations among the studied cognitive, behavioral, and psychosocial factors; the potential mediator (ie, negative psychological responses to COVID-19); and the dependent variable (ie, depression). Collinearity diagnosis of the aforementioned independent variables and mediators was conducted by examining the variance inflation factor (VIF); a VIF value greater than five would suggest existence of collinearity. By using structural equation modeling (SEM) with maximum likelihood estimation, the potential mediation and suppression effects of mental distress due to COVID-19 between the cognitive/behavioral/psychological factors and depression were tested, adjusting for all studied background personal and contextual variables. Three latent variables were created for the SEM analysis: perceived infection risk (derived from the original three items), mental distress due to COVID-19 (derived from the original three items), and depression (derived from three parcels that were randomly grouped from the original nine items). The random parceling approach has been recommended for SEM analysis [39]. Other independent variables were represented by single items. The recommended model fit index included the comparative fit index ≥0.90, the normed-fit index ≥0.90, the Tucker-Lewis index ≥0.90, and the root mean square error of approximation ≤0.08. The SEM was conducted using AMOS 17.0 (IBM Corp), while other analyses were performed using SPSS 21.0 (IBM Corp). The significance level was defined as a two-tailed *P*<.05.

## Results

### Descriptive Statistics

Descriptive statistics are presented in Table 1. Out the 23,863 responses for the cognitive variables, there was perceived airborne transmission (n=5590, 23.4%); a perceived high or very high risk of contracting COVID-19 for oneself (n=2672, 11.2%), family members (n=2814, 11.8%), or classmates (n=4367, 18.3%); perceived permanent bodily damage (n=8523, 35.7%); and a perceived high chance of controlling COVID-19 in China in the coming 6 months (n=16,714, 70.0%). Behaviorally, 49.3% (n=11,757) stayed at home all the time during the 7-day CNY period (the modal response); 4.9% (n=1159) reported that they had close contact with people who visited Wuhan 2 weeks prior to the CNY. Regarding the psychosocial factor, 21.5% (n=5124) perceived high or very high levels of discrimination due to COVID-19.

Regarding psychological responses, 47.1% (n=11,235) reported high or very high levels of one or more types of mental distress, panic (n=9483, 39.8%), anxiety (n=8483, 35.5%), or emotional agitation (n=8045, 33.7%) due to the COVID-19 epidemic; the composite variable of mental distress due to COVID-19 summed up the item scores of these three types of responses. The mean was 6.9 (SD 2.2, range 3-12). Furthermore, about 40% of the participants showed mild to severe depression (mild: n=5862, 24.6%; moderate or severe: n=3464, 14.5%). The mean of the PHQ-9 score was 4.6 (SD 5.5, range 0-27; see Table 2).

**Table 1:** Background variables of the participants (N=23,863).

Variables					Participants, n (%)
**Sociodemographics**					
	**Sex**				
		Male		7605 (31.9)	
		Female		16,258 (68.1)	
	**School-related information**				
		**Grade**			
			First year	9017 (37.8)	
			Second year	6425 (26.9)	
			Third year	5061 (21.2)	
			Fourth year	2281 (9.6)	
			Fifth year	542 (2.3)	
			Master’s or above	537 (2.3)	
		**Faculty**			
			Medicine	10,850 (45.5)	
			Arts	4232 (17.7)	
			Science	3901 (16.4)	
			Engineering	1809 (7.6)	
			Social science	846 (3.6)	
			Others	2225 (9.3)	
	**Living arrangement during CNY^a^**				
		**Staying in the university’s city**			
			No	11,116 (46.6)	
			Yes	12,747 (53.4)	
		**Staying with family**			
			No	1559 (6.5)	
			Yes	22,304 (93.5)	
	**Self-reported physical health status**				
		Moderate/poor/very poor		4926 (20.6)	
		Good/very good		18,937 (79.4)	
**Information about participants’ localities of stay at the time of survey**					
	**Local entry/exit control during CNY (shutdown)**				
		No		7018 (29.4)	
		Yes		16,845 (70.6)	
	**Confirmed COVID-19 cases^b^ in the province participant was in**				
		0-50		4965 (20.8)	
		51-150		8385 (35.1)	
		151-300		5581 (23.4)	
		>300		4932 (20.6)	

^a^CNY: Chinese Lunar New Year.

^b^The number of cumulative confirmed COVID-19 cases refers to the national data reported by the launch day of this study (February 1, 2020).

**Table 2:** Descriptive statistics of the independent variables, the mediator, and the dependent variable (N=23,863).

Variables				Participants
**Cognitive factors, n (%)**				
	**Perceived airborne transmission**			
		No/do not know	18,273 (76.6)	
		Yes	5590 (23.4)	
	**Perceived Infection Risk Indicator^a^**			
		0	18,779 (78.7)	
		1	1846 (7.7)	
		2	1707 (7.2)	
		3	1531 (6.4)	
	**Perceived permanent bodily damage**			
		Disagree/do not know	15,340 (64.3)	
		Agree	8523 (35.7)	
	**Perceived chance of controlling the epidemic within 6 months**			
		Else	7149 (30.0)	
		Definitely yes/very high	16,714 (70.0)	
**Behavioral factors, n (%)**				
	**Time spent going out during CNY^b^ (hours)**			
		≥15	1295 (5.4)	
		11-14	1233 (5.2)	
		5-10	4156 (17.4)	
		1-4	5422 (22.7)	
		0	11,757 (49.3)	
	**Close contacts with people who had visited Wuhan 2 weeks before CNY**			
		No or do not know	22,704 (95.1)	
		Yes	1159 (4.9)	
**Psychosocial factors, n (%)**				
	**Perceived discrimination due to COVID-19**			
		Very low	9989 (41.9)	
		Low	8750 (36.7)	
		High	3690 (15.5)	
		Very high	1434 (6.0)	
**Mental distress to COVID-19, mean (SD)**				6.9 (2.2)
	0, n (%)			12,628 (52.9)
	1, n (%)			2892 (12.1)
	2, n (%)			1910 (8.0)
	3, n (%)			6433 (27.0)
**Depression (PHQ-9^c^), mean (SD)**				4.6 (5.5)
	Normal, n (%)			14,537 (60.9)
	Mild, n (%)			5862 (24.6)
	Moderate, moderately severe, or severe, n (%)			3464 (14.5)

^a^The Perceived Infection Risk Indicator counted the number of endorsements of “high/very high” for three items about perceived risk of infection for oneself, family members, and classmates; the indicator counted the number of endorsements of “high/very high” for three items measuring negative psychological responses to COVID-19; details are described in the Methods section.

^b^CNY: Chinese Lunar New Year.

^c^PHQ-9: Patient Health Questionaire-9.

### Associations Between Background Variables and Depression

The associations between background variables and depression are presented in Table 3. Females showed more depressive symptoms than males, but the difference did not reach statistical significance (*P*=.07). Several contextual factors were significantly associated with lower risks of depression, including staying in the city of the university, staying with family, and self-perceived physical health, while the number of confirmed cases (>300 cases) detected in the province where the participants were staying at the time of the survey was positively associated with depression symptoms (ie, higher scores of PHQ-9). Whether the city had been *shut down* was, however, not associated with depression. The background variables were adjusted for in the SEM analysis.

**Table 3:** Linear regression analyses on the associations between background variables and depression (n=23,863).

Variables					Depression		
					β		*P* value
**Sociodemographics**							
	**Sex**						
		Male			—^a^		—
		Female			–.01		.07
	**School-related information**						
		**Grade**					
			First year	—		—	
			Second year	.02		.005	
			Third year	–.01		.11	
			Fourth year	–.01		.51	
			Fifth year	–.01		.41	
			Master’s or above	.01		.40	
		**Faculty**					
			Medicine	—		—	
			Arts	.04		<.001	
			Science	.01		.04	
			Engineering	.01		.16	
			Social science	.02		.009	
			Others	–.01		.27	
	**Living arrangement during CNY^b^**						
		**Staying in the university’s city**					
			No	—		—	
			Yes	–.02		.02	
		**Staying with family**					
			No	—		—	
			Yes	–.04		<.001	
	**Self-reported physical health status**						
		Moderate/poor/very poor			—		—
		Good/very good			–.28		<.001
**Information about participants’ localities of stay at the time of survey**							
	**Local entry/exit control during CNY (shutdown)**						
		No			—		—
		Yes			–.01		.85
	**Confirmed COVID-19 cases^c^ in the province participant was in**						
		0-50			—		—
		51-150			.01		.48
		151-300			.01		.04
		>300			.03		<.001

^a^Reference variable.

^b^CNY: Chinese Lunar New Year.

^c^The number of cumulative confirmed COVID-19 cases refers to the national data reported on the launch day of this study (February 1, 2020).

### The Mediation Analysis

#### Correlations Among Variables

A number of variables were positively correlated with depression, including the Perceived Infection Risk Indicator (*r*=0.11, *P*<.001), having close contacts with people who had visited Wuhan 2 weeks prior to the CNY (*r*=0.05, *P*<.001), perceived discrimination due to COVID-19 (*r*=0.14, *P*<.001), and mental distress due to COVID-19 (*r*=0.25, *P*<.001). Some variables were negatively correlated with depression, including perceived chance of getting the epidemic under control in China within 6 months (*r*=–0.13, *P*<.001) and staying at home (*r*=–0.08, *P*<.001). Perceived airborne transmission (*r*=–0.01, *P*=.29) and perceived permanent bodily damage (*r*=.01, *P*=.44) were *not* significantly associated with depression; mediation analyses were hence not performed for these two associations (see Table 4).

**Table 4:** Spearman correlations among the independent variables, the mediator, and depression (N=23,863).

Variables		Depression	
		*r*	*P* value
**Cognitive factors**			
	Perceived airborne transmission	–0.01	.29
	Perceived Infection Risk Indicator^a^	0.11	<.001
	Perceived permanent bodily damage	0.01	.44
	Perceived chance of controlling the epidemic within 6 months	–0.13	<.001
**Behavioral factors**			
	Staying at home	–0.08	<.001
	Close contacts with people who had visited Wuhan 2 weeks before CNY^b^	0.05	<.001
**Psychosocial factors**			
	Perceived discrimination due to COVID-19	0.14	<.001
**Mediating variables**			
	Mental distress due to COVID-19 (the summative score)	0.25	<.001

^a^The Perceived Infection Risk Indicator counted the number of endorsements of “high/very high” for three items about perceived risk of infection for oneself, family members, and classmates; the indicator counted the number of endorsements of “high/very high” for three items measuring negative psychological responses to COVID-19; details are described in the Methods section.

^b^CNY: Chinese Lunar New Year.

#### Testing Mediation and Suppression Effects of Negative Psychological Responses Between the Studied Factors and Depression

**Figure 1:**
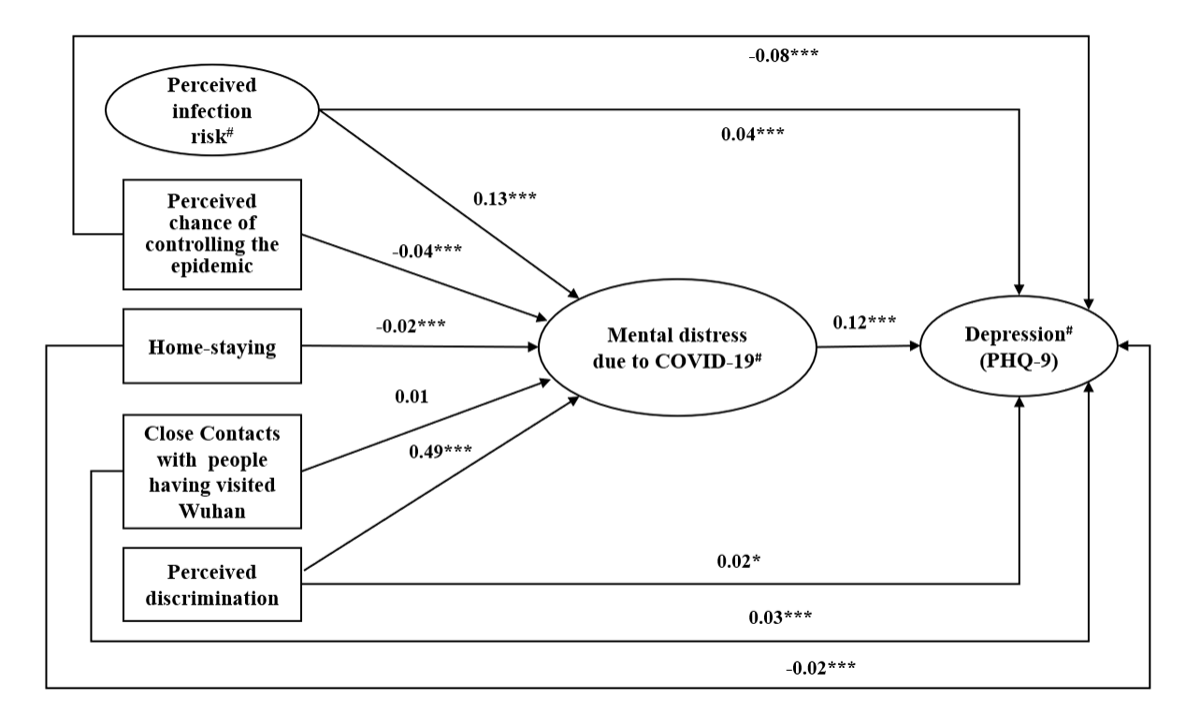
The mediation effect of mental distress due to COVID-19 on the associations between the independent variables and depression. The structural equation model was adjusted for background variables, including sex, school-related information, living arrangement during the Chinese Lunar New Year, self-reported physical health status, and information about localities of stay at the time of the survey. # indicates latent variables; details are described in the Methods of the text. PHQ-9: Patient Health Questionnaire-9. *P<.05; **P<.01; ***P<.001.

The SEM model’s fit index was satisfactory (comparative fit index 0.95, normed-fit index 0.94, Tucker-Lewis index 0.94, and root mean square error of approximation 0.04); the range of factor loadings for the three latent variables was 0.70-0.97 (all *P*<.001). No collinearity was detected with the VIF values of all studied variables ranging from 1.00 to 1.37 (VIF>5 indicates the existence of collinearity). In Figure 1, mental distress due to COVID-19 partially mediated or suppressed the association between perceived infection risk and depression (mediation effect size 27.4%, Sobel test *P*<.001), between perceived discrimination and depression (mediation effect size 79.5%, Sobel test *P*<.001), between perceived chance of epidemic control and depression (suppression effect size 6.0%, Soble test *P*<.001), and between staying at home behavior and depression (suppression effect size 9.8%, Sobel test *P*<.001). The nonsignificant mediator was close contacts with people who had visited Wuhan (Sobel test *P*=.32). The beta values are shown in Figure 1.

## Discussion

This population-based study, which covers 26 universities of 16 cities in 13 provinces of China, revealed a relatively high level of depressive symptoms among university students during the initial phase of the COVID-19 outbreak in China (10-20 days since the official recognition of the person-to-person transmission property of COVID-19). This study identifies a number of significant COVID-19–related cognitive (ie, perceived infection risk and perceived controllability of the epidemic), behavioral (ie, staying at home behavior and close contacts with people from Wuhan), and psychosocial (ie, perceived discrimination) factors of both mental distress due to COVID-19 and depressive symptoms; nonsignificant factors included perceived airborne transmission and perceived permanent bodily damage. As expected, mental distress due to COVID-19 was significantly and positively associated with depressive symptoms; it further mediated the associations between some of the cognitive/behavioral/psychosocial factors and depressive symptoms. The findings present a rough snapshot of what happened in the country and shed light on how people may react emotionally to new emerging infectious diseases and how various types of responses (eg, cognitive and behavioral responses) would be associated with such emotional responses.

The COVID-19 pandemic has affected people’s mental health. The findings of this study reveal prevalent mild to severe depression in 40% (9326/23,863) of university students in China, according to the PHQ-9. The prevalence obtained from studies among university students in China prior to the COVID-19 period seemed lower (eg, 23.8% among Chinese university students reported in a meta-analysis [40] and 29.5% reported during the SARS outbreak among Chinese university students [41]), but such prevalence was not exactly comparable, as different tools and sampling methods were used. Three of the four studies on Chinese university students conducted during a similar time period also used the PHQ-9. Two of the studies, which were conducted in Guangzhou [11] and 29 Chinese provinces [14], presented prevalence of moderate to severe depression (PHQ-9≥10) of 7.7% and 23.3%, respectively, compared to the 14.5% (3464/23,863) of this study, denoting geographical variations in addition to differences in sampling methods. This study has the strength that it was population-based and had a class-based sampling frame, while the others were distributed conveniently online.

Depressive symptoms were prevalent among university students at the initial phase of the COVID-19 outbreak in China; this can be seen from the high levels of self-reported mental distress directly attributed to COVID-19, which is understandable, as uncertainties and the Wuhan lockdown were alarming and worrisome. Furthermore, mental distress due to COVID-19 was positively associated with depressive symptoms. Thus, the mental distress directly attributed to COVID-19 might have increased the already high level of depression among university students during the initial phase of the epidemic in China. As mental distress and depression affect preventive behaviors [9] and individual well-being [42], and acute stress may turn into chronic depression [43,44], health care workers need to integrate mental health promotion with prevention of COVID-19 at the initial stages of outbreaks for new emerging infectious diseases.

The context of this study was unique and relevant, as it was conducted during the CNY and soon after the first outbreak and lockdown in Wuhan. It can be seen that students who stayed in the *university’s city* or with family members were less likely than others to be depressed. They might have received better support from their significant others, and social support and coping resources are protective against mental health problems [45,46]. The perceived number of confirmed cases in the *province they were located in* was positively associated with depression; it is plausible that the perception might increase perceived susceptibility and perceived severity of the epidemic and thus depression. It is interesting that travel restrictions on entering and exiting from the city of stay, which was then a *new* preventive measure, was not significantly associated with depression. The findings suggest that such drastic restriction, if implemented orderly and with good support, does not necessarily cause substantial panic or negative impacts on mental distress. A number of countries implemented even more severe lockdowns such as bans on going out soon after the completion of this study (eg, Italy), which was followed by many other countries (eg, parts of the United Kingdom, France, and Australia). In some countries, the strict social distancing measures were associated with depression [27,47]. Future studies should review this *new* measure and identify ways to minimize its adverse mental health effects. This study hence documents the initial responses to preliminary lockdown measures in the COVID-19 pandemic.

This study has interesting findings that involve interpretations in the context of the initial outbreak and in comparison with other studies. Spearman analysis and the SEM showed that some COVID-19–related cognitions (perceived chance of controlling COVID-19 and perceived risks of infection) were significantly associated with both mental distress due to COVID-19 and depressive symptoms; such findings corroborate with other studies [15,20-23]. However, it is unexpected that perceived bodily damages (a reflection of perceived severity) and perceived airborne transmission (possibly a misconception) were not significantly correlated with mental distress due to COVID-19 and depression, while such correlations were significant in similar H1N1 research [7,8]. It is plausible that this study was conducted in the early prepandemic phase of COVID-19 when no clear information was given about long-term harms and modes of transmission.

The behavioral factors of self-reported mental distress due to COVID-19 and depressive symptoms also illustrated the uniqueness of this study. The duration of staying at home during the CNY was protective against depression, which has not been reported in the global literature (except one general population study that found a nonsignificant result [15]). However, other studies have reported positive associations between social distancing and isolation and depression [27,47]. It has some special implications, as there were then no clear and strict social distancing policies in China (except in Wuhan), and people could leave home without facing penalties. The government, however, pledged for national support to contain COVID-19; staying at home during the CNY hence might have involved altruism and social responsibility, which were negatively associated with depression [48]. People may also feel safer at home. Thus, a short period of staying at home (for the 7-day holiday) during the initial outbreak period of a new emerging infectious disease may not cause mental distress but was instead protective. It seems that social distancing policies need to be exercised as early as possible during new outbreaks of important emerging infectious diseases to increase effectiveness and minimize distress. In addition, we found that close contacts with people coming from Wuhan was a risk factor of depression. To our knowledge, no study has looked at this variable, although other studies looked at visits to Wuhan (eg, [13]). This finding has important implications, as Wuhan was the epicenter where the first outbreak occurred, and the virus was spread to other regions. The variable became nonsignificant in the SEM, possibly because of controlling for a potential confounder of perceived discrimination.

This study also investigated the psychosocial factor of perceived discrimination. Over half (13,874/23,863, 58.1%) of the participants perceived discrimination related to COVID-19, possibly because of their traveler status. Associations between perceived discrimination and depression were similarly reported in previous studies related to SARS and H1N1 [7,8,49]. In fact, the association with depression was the strongest one among all the factors of this study. Thus, university students may feel more depressed during the COVID-19 epidemic, not only because of related perceived susceptibility and severity but also the way they are treated by others. To our knowledge, only one Canadian general population study had looked at such an association but found a nonsignificant finding [16]. The situation in China was unique. The country faced strong international pressure during the study period; Wuhan was accused of spreading the disease to other regions. Indeed, COVID-19 was initially labelled as the *Wuhan virus*. Travelers might be regarded as potential carriers of the virus; discrimination found a fertile ground to grow and might have a powerful negative effect on mental distress. It is imperative to investigate whether perceived discrimination related to epicenters has caused mental distress in the later phase of the pandemic. For instance, there were over 22 million detected cases in the United States as of January 10, 2021, and a new viral strain of higher infectivity was found in the United Kingdom where the incidence of COVID-19 is soaring. The level of perceived discrimination and its association with depression might be country specific due to politicization. When facing outbreaks of new emerging infectious diseases in the future, stigma needs to be removed from the location of the outbreak. The effects of the generalized perceived discrimination need to be investigated.

This is one of the few studies that looked at the mechanisms behind the associations between COVID-19–related factors and depression during the COVID-19 period. The findings suggest that COVID-19–related cognitive, behavioral, and psychosocial factors, and mental distress directly attributed to COVID-19 were all associated with depression, while the relationships between some of the COVID-19 cognitive, behavioral, and psychosocial factors, and depression may be partially mediated and suppressed through mental distress due to COVID-19. Specifically, perceived risk and perceived discrimination may have an indirect effect on depression via mental distress due to COVID-19; such risk factors might have increased mental distress due to COVID-19, which would in turn increase risk of depression. In addition, confidence in controlling the epidemic and staying at home could potentially be protective against depression via reduction of mental distress due to COVID-19, which was in turn positively associated with depression. Interventions to improve these cognitions or preventive behaviors may thus reduce depression directly or via reduction of mental distress due to COVID-19. Moreover, the mediation and suppression model presented in Figure 1 are supported by the common sense model [36], which suggests that diseases (COVID-19 in this case) as stimuli trigger cognitive representations (perceptions related to COVID-19) and emotional representations (negative psychological responses to COVID-19). The two types of responses would in parallel determine the coping process and health outcomes (depression in this case). In addition, the theory postulates that the cognitive responses would have an effect on the emotional responses. The findings and the model suggest that both cognitive and emotional outcomes are important in jointly determining mental distress during the COVID-19 period. No study has tested this contention. Future longitudinal studies are warranted to test the full common sense model in the context of the COVID-19 epidemic.

This study has the strength of covering a large number of university students who were staying in most of the provinces in China. The data thus presents a crude *national* scenario. This study has some limitations. First, it did not have national coverage. Selection bias may exist, as classes and departments were not randomly selected. Second, we did not cover important interpersonal factors (eg, subjective norms and social support), which were associated with many health-related behaviors [50,51]. Third, the relatively mild magnitudes of some mediation and suppression effects of mental distress due to COVID-19 imply existence of other unstudied mechanisms. Last, the cross-sectional study design does not allow for causal inferences, as depression may also change perceptions. Longitudinal studies are needed to confirm these contentions.

The findings suggest that mental distress due to COVID-19 and depression were prevalent among university students in China during the initial COVID-19 outbreak period. The former may have further increased the prevalence of the latter. Various cognitive, behavioral, and psychosocial responses to COVID-19 showed both direct and indirect effects (via mental distress due to COVID-19) on depression. Thus, interventions to improve such multidimensional factors might reduce mental distress during the initial COVID-19 outbreak period. The associations between some of the studied factors and depression may change over time as more information and experiences were obtained by the public, signifying early investigation of community responses to avoid mental distress, which would carry over to later phases and affect prevention behaviors. Some of the findings may shed light on handling new emerging infectious diseases that occur in the future. It is important to validate the findings in general and specific populations in China and in other countries.

## Abbreviations

CNY: Chinese Lunar New Year
H1N1: novel influenza A
PHQ-9: Patient Health Questionnaire-9
QR: Quick Response
SARS: severe acute respiratory syndrome
VIF: variance inflation factor
